# Neuromusculoskeletal model-informed machine learning-based control of a knee exoskeleton with uncertainties quantification

**DOI:** 10.3389/fnins.2023.1254088

**Published:** 2023-08-30

**Authors:** Longbin Zhang, Xiaochen Zhang, Xueyu Zhu, Ruoli Wang, Elena M. Gutierrez-Farewik

**Affiliations:** ^1^KTH MoveAbility Lab, Department of Engineering Mechanics, KTH Royal Institute of Technology, Stockholm, Sweden; ^2^Department of Mathematics, University of Iowa, Iowa City, IA, United States

**Keywords:** machine learning, data-driven biomechanical models, inverse dynamics, neuromusculoskeletal modeling, uncertainty quantification

## Abstract

**Introduction:**

Research interest in exoskeleton assistance strategies that incorporate the user's torque capacity is growing rapidly. However, the predicted torque capacity from users often includes uncertainty from various sources, which can have a significant impact on the safety of the exoskeleton-user interface.

**Methods:**

To address this challenge, this paper proposes an adaptive control framework for a knee exoskeleton that uses muscle electromyography (EMG) signals and joint kinematics. The framework predicted the user's knee flexion/extension torque with confidence bounds to quantify the uncertainty based on a neuromusculoskeletal (NMS) solver-informed Bayesian Neural Network (NMS-BNN). The predicted torque, with a specified confidence level, controlled the assistive torque provided by the exoskeleton through a TCP/IP stream. The performance of the NMS-BNN model was also compared to that of the Gaussian process (NMS-GP) model.

**Results:**

Our findings showed that both the NMS-BNN and NMS-GP models accurately predicted knee joint torque with low error, surpassing traditional NMS models. High uncertainties were observed at the beginning of each movement, and at terminal stance and terminal swing in self-selected speed walking in both NMS-BNN and NMS-GP models. The knee exoskeleton provided the desired assistive torque with a low error, although lower torque was observed during terminal stance of fast walking compared to self-selected walking speed.

**Discussion:**

The framework developed in this study was able to predict knee flexion/extension torque with quantifiable uncertainty and to provide adaptive assistive torque to the user. This holds significant potential for the development of exoskeletons that provide assistance as needed, with a focus on the safety of the exoskeleton-user interface.

## 1. Introduction

Exoskeletons have enormous potential to enhance movement and to contribute to neuromuscular rehabilitation in persons with motor disorders such as stroke, cerebral palsy, and spinal cord injury (Sartori et al., 2016; Li et al., 2019, 2021; Liu et al., 2019; Zhang et al., 2019). Exoskeleton-assisted rehabilitation training involves the use of control algorithms aimed at improving muscle strength, neuroplasticity, and movement enhancement in users (Fujii et al., 2017). These control strategies can be classified into three types: passive control, triggered passive control, and assist-as-needed control (Marchal-Crespo and Reinkensmeyer, 2009; Meng et al., 2015; Proietti et al., 2016). Passive control refers to a technique in which the exoskeleton is in charge and guides the user to follow predefined trajectories or assistive forces/torques that have been extracted from healthy populations. The user is passive in the movement and does not actively control the exoskeleton. This type of control is often used in the initial stages of therapy to re-acquaint a limb to movement. Triggered passive control is a variant of passive control, where the user initiates the exoskeleton's assistance. Once activated, the user is again passive in the movement as the exoskeleton moves along pre-determined trajectories. This technique is often used to incorporate the brain-machine interface into the control process, providing assistance to individuals with irreversible impairments, such as tetraplegia (Proietti et al., 2016). Assist-as-needed control, also known as “user-in-charge” or “active control,” empowers the user to perform daily tasks with the aid of an exoskeleton. The exoskeleton provides assistance based on the user's ability and intention to generate torque, with the aim of promoting neuroplasticity and user autonomy. This active control technique is typically applied in persons with residual motor function (Chen et al., 2016; Durandau et al., 2017; Li et al., 2018; Yao et al., 2018). The primary focus of this paper is on active control techniques that seek to supplement the user's insufficient muscle contributions with assistance from an exoskeleton. Providing torque assistance based on the user's movement intention requires precise and robust decoding of motor function, which can be achieved through recording of underlying neuromuscular activities, such as brain and nerve signals and muscle electromyography (EMG) signals. EMG signals, which capture the electrical excitation of muscles, are a commonly used method for predicting joint torques, as they are easy to obtain and offer crucial insights into human motion (Sartori et al., 2018; Huang et al., 2019; Mounis et al., 2019).

Joint torque prediction is crucial in the control of exoskeleton-assisted rehabilitation and has frequently been achieved through two methods: physics-based neuromusculoskeletal (NMS) modeling and artificial neural networks (ANNs) (Pizzolato et al., 2015, 2019; Zhang et al., 2020, 2021). To improve prediction accuracy, ANNs have been integrated into NMS models in recent research. In our recent study (Zhang et al., 2022), an NMS solver-informed ANN model was developed to estimate ankle joint torque by combining features from an NMS model with a standard ANN, based on measured joint angles and muscle EMG signals during gait and isokinetic motions. This hybrid model was overall more accurate than the NMS or standard ANN models alone, but still showed poor prediction performance in one subject during gait, possibly due to incorporating less informative or misleading input features from the NMS model. This highlights the necessity of quantifying the uncertainty of joint torque predictions for safe and efficient human-exoskeleton interaction; accurate estimation of joint torque is crucial for determining the appropriate level of assistance from an exoskeleton.

A Bayesian Neural Network (BNN) is a well-established type of ANN for making predictions with uncertainties and has great potential in safe and efficient exoskeleton control (Cursi et al., 2021; Wei et al., 2021; Zhong et al., 2021). Unlike conventional ANNs (Cao et al., 2022b; Hu et al., 2022), BNNs incorporate probability distributions to represent prediction uncertainty and provide a probability distribution indicating the likelihood of different outcomes (Cursi et al., 2021). This characteristic makes BNNs useful for decision-making in various fields, including biomechanics, meteorology, and robotics. For instance, Zhong et al. (2021) developed a BNN-based framework for predicting the environmental context of lower limb prostheses. The quantified prediction uncertainty could lead to context recognition strategies that enhance reliable decision-making, efficient sensor fusion, and improved design of intelligent systems for various applications. Another popular technique for making predictions with uncertainties is the Gaussian Process (GP) (Chen et al., 2013; Yun et al., 2014; Maritz et al., 2018; Guo et al., 2019; Cao et al., 2022a). GP models the output as a Gaussian distribution with mean and covariance parameters, wherein the uncertainty is expressed by the covariance. Liang et al. (2021), for example, developed a GP model to estimate knee joint angles and uncertainties from EMG signals during walking and running movements. As both BNNs and GPs can estimate prediction uncertainty, it is of interest to compare the two methods in the context of safe and efficient human-exoskeleton interaction.

The objective of this study was thus to develop an NMS uncertainty-informed adaptive control framework for a knee exoskeleton. The framework aimed to provide accurate predictions of the user's knee flexion/extension (F/E) physiological torque, while also quantifying the level of estimation uncertainty. To achieve this, an NMS solver was employed to inform the machine learning models, which would subsequently adjust the assistance level based on the level of uncertainty. Another aim was to compare the predictions with uncertainties from the NMS solver-informed BNN (NMS-BNN) model with those from the NMS solver-informed GP (NMS-GP) model.

## 2. Methods

**Figure 1:**
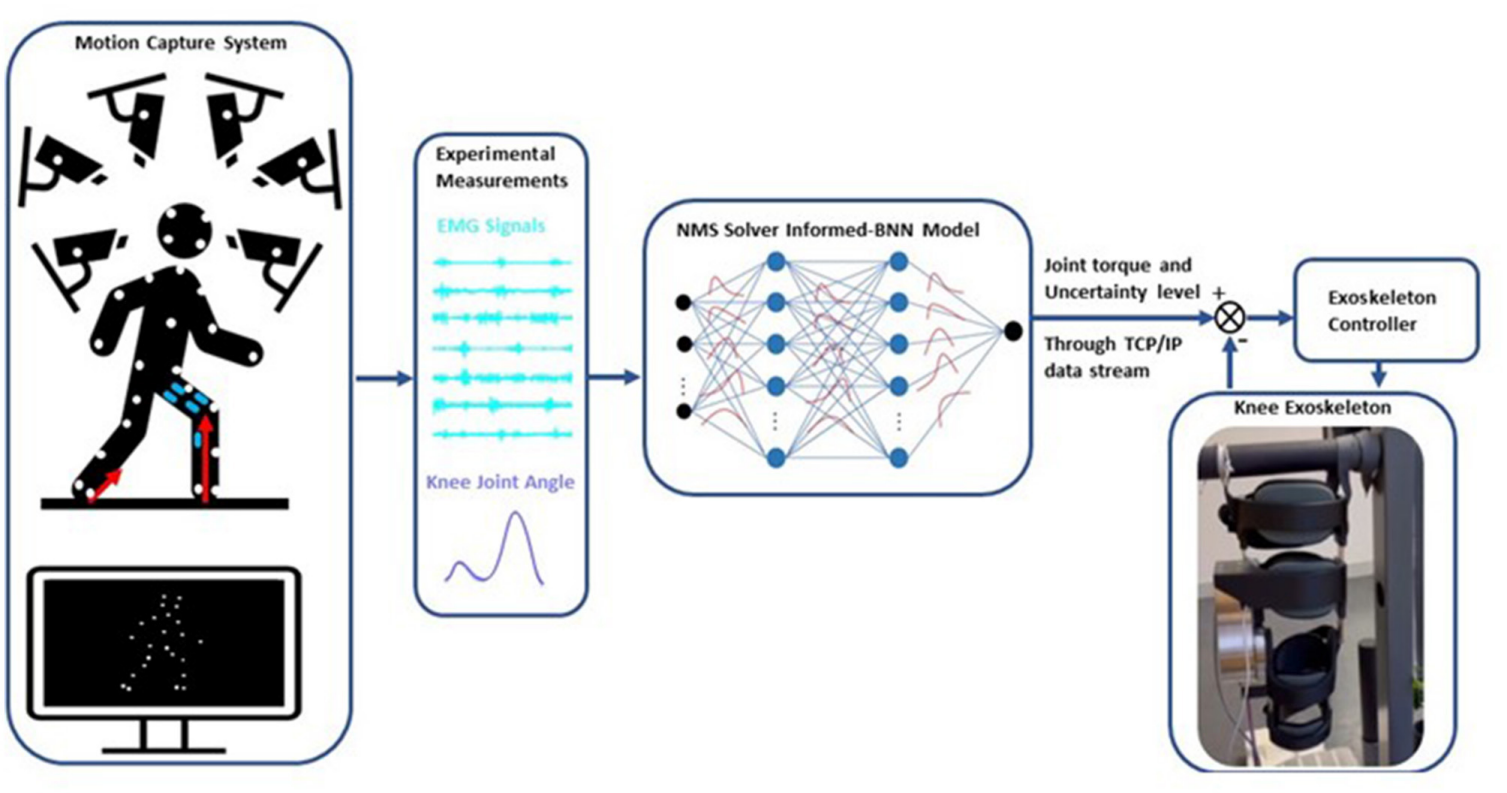
Schematic of the adaptive control framework for a knee exoskeleton based on an NMS solver-informed BNN (NMS-BNN) model. The inputs to the NMS-BNN include observed muscle signals and joint angles, as well as physical features derived from the NMS solver such as individual muscle force and joint torque. The NMS-BNN outputs knee joint torque with uncertainty quantification in the form of confidence bounds. The predicted torque with a specified confidence level is then used to control the assistive torque provided by the knee exoskeleton through a TCP/IP data stream.

We developed an adaptive control framework for a knee exoskeleton based on an NMS-BNN model (Figure 1). The NMS-BNN takes two types of inputs: (1) experimental measurements—muscle signals and joint angles, and (2) informative physical features extracted from the underlying NMS solver, such as individual muscle force and joint torque. The NMS-BNN outputs knee joint torque with uncertainty quantification in the form of confidence bounds. The predicted torque with a specified confidence level is then used to control the assistive torque provided by the knee exoskeleton through a TCP/IP data stream. The study results consist of two key components: (1) an analysis of the NMS-BNN model's prediction accuracy and uncertainty, compared to the traditional NMS model and to the NMS-GP model; (2) an evaluation of the tracking performance of the assistive torque provided by the knee exoskeleton.

### 2.1. Data collection and processing

**Figure 2:**
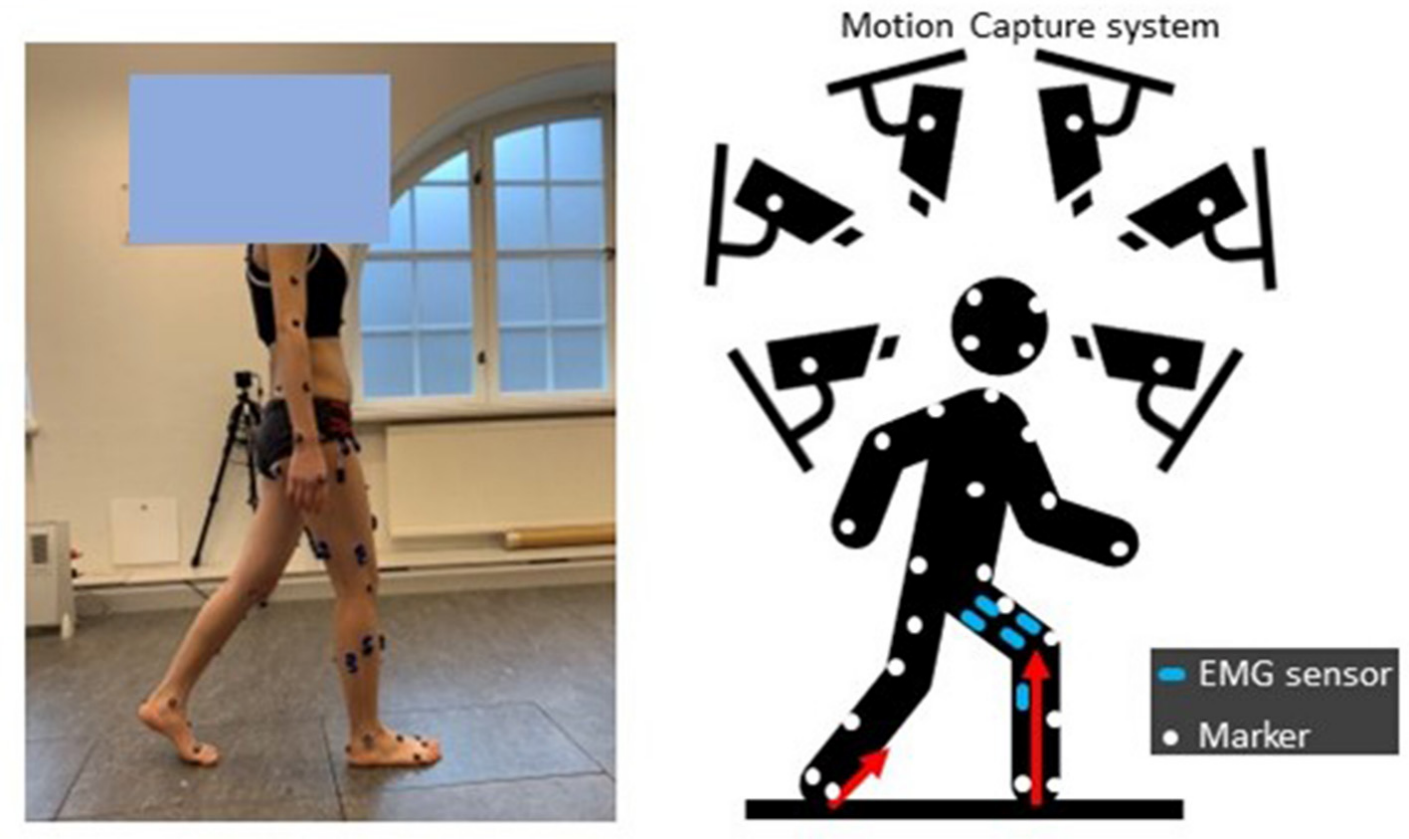
Experimental setup: subjects equipped with EMG sensors and markers, performed movements in an instrumented motion lab.

Eight able-bodied subjects (sex: 4F/4M; height: 168.1 ± 9.4 cm; weight: 65.2 ± 17.8 kg; age: 29 ± 4 years) were recruited. The Swedish Ethical Review Authority (Dnr. 2020-02311) approved this study, and all subjects provided informed written consent documents. All participants were asked to do five movement types (Figure 2), specifically slow walking, normal walking, fast walking, sit-to-stand, and stand-to-sit. During the experiments, each movement was repeated at least ten times. The sequence of movements was randomized.

Surface EMG signals (aktos nano, myon, Schwarzenberg, Switzerland) from vastus medialis (VM), vastus lateralis (VL), rectus femoris (RF), semitendinosus (ST), biceps femoris (BF), gastrocnemius medialis (GM), and gastrocnemius lateralis (GL) of each participant's one randomly-selected leg were measured at 1,000 Hz. EMGs were post-processed by bandpass filtering (30–300 Hz), rectifying, low pass filtering (6 Hz), and normalizing to the maximum EMG value among all movement trials (Sartori et al., 2016; Pizzolato et al., 2017; Hoang et al., 2018).

Marker trajectories were recorded at 100 Hz using a 3D motion capture system (V16, Vicon, Oxford, UK), with marker placement based on the CGM2.3 model (Leboeuf et al., 2019). Ground reaction forces (GRFs) were measured at 100 Hz with three force plates (AMTI, MA, USA). Kinematics were calculated via inverse kinematics by solving a weighted least square optimization problem to minimize the discrepancy between virtual *x*_*i*_ and measured $x_{i}^{exp}$ marker trajectories (Lu and O'connor, 1999), Equation (1).


(1) $$\begin{array}{l} \underset{q}{\min}\left(\overset{N}{\underset{i}{\sum }}\theta_{i}||x_{i}^{exp}-x_{i}|{|}^{2}\right), \end{array}$$

where *q* represents the generalized coordinates of the model and θ_*i*_ is the weight of *i*th marker. Kinetics were computed via inverse dynamics by solving for joint torques in the dynamic equations of motion (Pandy, 2001) (Equation 2),


(2) $$M(q)\ddot{q}+C(q,\dot{q})+G(q)+R(q)F^{mt}+F_{e}=0$$

where $\text{q},\dot{q},\ddot{q}$ are the vector of generalized position, velocity and acceleration, respectively; *M*(**q**) is mass matrix and $M\left(\text{q}\right)\ddot{q}$ is a vector of inertial forces and torques; $C\left(q,\dot{q}\right)$ is the vector of centripetal and Coriolis forces and torques; **G**(**q**) is the vector of gravitational forces and torques; *R*(**q**) is the matrix of muscle moment arms; **F**^**mt**^ is a vector of musculotendon forces and *R*(**q**)**F**^**mt**^ is the vector of musculotendon torques; **F**_**e**_ is the vector of external force and torques (i.e., GRFs in this paper). A low-pass fourth-order zero-lag Butterworth filter (6 Hz) was used to filter joint kinematics and kinetics (Winter et al., 1974; Mantoan et al., 2015; Derrick et al., 2020).

### 2.2. EMG-driven neuromusculoskeletal model

**Figure 3:**
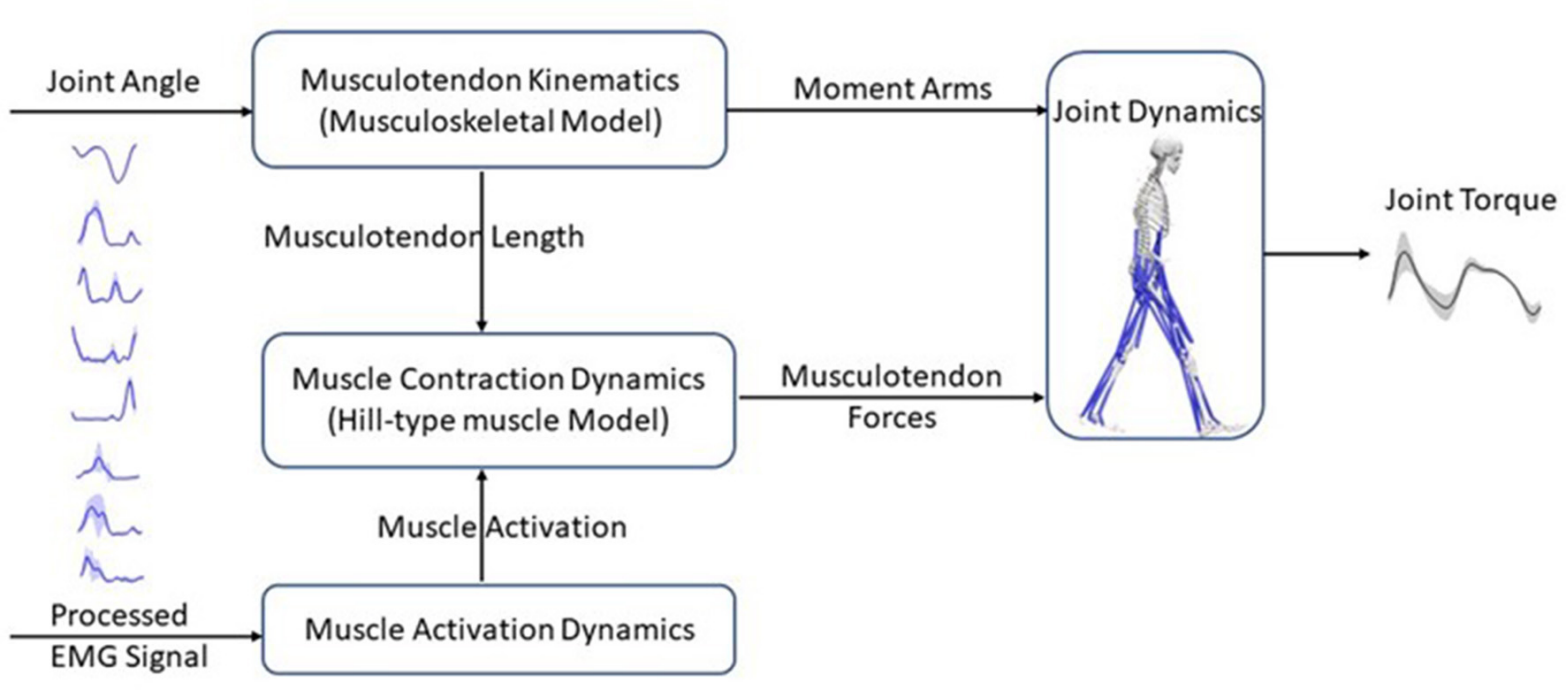
Schematic structure of an EMG-driven neuromusculoskeletal model with four components: the musculotendon kinematics component calculates musculotendon lengths and moment arms; the muscle activation dynamics component uses the EMG information to compute muscle activation; the muscle contraction dynamics component, predicts musculotendon force using musculotendon length and muscle activation based on a Hill-type muscle model; and finally, the joint dynamics component computes joint torques with musculotendon forces and moment arms as inputs.

The EMG-driven NMS model used in this study was the open-source CEINMS model (Pizzolato et al., 2015) (Figure 3). This model includes four components: musculotendon kinematics, muscle activation dynamics, muscle contraction dynamics, and joint dynamics relationships (Sartori et al., 2011). The musculotendon kinematics component calculates moment arms and musculotendon lengths, while the muscle activation dynamics component computes muscle activation based on the available EMG information. The relationship between EMG excitation, *e*(*t*), and neural activation, *u*(*t*), is expressed in Equation (3) (Lloyd and Besier, 2003):


(3) $$\begin{array}{l} u\left(t\right)=\alpha \cdot e\left(t-\tau \right)-\beta_{1}\cdot u\left(t-1\right)-\beta_{2}\cdot u\left(t-2\right) \end{array}$$

where α is the muscle gain parameter, β_1_ and β_2_ are the recursive parameters [β_1_ = *C*_1_+*C*_2_, β_2_ = *C*_1_·*C*_2_, with |*C*_1_| < 1, |*C*_2_| < 1, and α−β_1_−β_2_ = 1 for a stable solution (Lloyd and Besier, 2003; Buchanan et al., 2004; Pizzolato et al., 2015)], and τ is the electromechanical delay. Muscle activation, *a*(*t*), is described by Equation (4):


(4) $$\begin{array}{l} a\left(t\right)=\frac{e^{Au\left(t\right)}-1}{e^{A}-1} \end{array}$$

where *A* is the shape factor (Buchanan et al., 2004; Hoang et al., 2018).

The muscle contraction dynamics component calculates the muscle force with a Hill-type muscle model, represented by Equation (5):


(5) $$\begin{array}{l} F=F_{0}^{m}\left[F_{al}\left(l\right)\cdot F_{v}\left(v\right)\cdot a+F_{pl}\left(l\right)+d_{p}\cdot v\right]cos\left(\theta \right) \end{array}$$

where $F_{0}^{m}$ is the muscle's maximum isometric force, *F*_*al*_(*l*) describes the relationship between active muscle force and fiber length *l*, *F*_*pl*_(*l*) describes the relationship between passive muscle force and fiber length, *F*_*v*_(*v*) describes the relationship between muscle force and fiber contraction velocity *v*, θ is the fiber pennation angle, and *d*_*p*_ is the muscle damping parameter.

Finally, the joint dynamics component computes joint torque by multiplying muscle forces and moment arms.

The parameters were calibrated as outlined by Pizzolato et al. (2015), with optimal fiber length and tendon slack length adjusted within ±15% of initial values, coefficients *C*_1_ and *C*_2_ limited to values between −1 and 1, and parameter *A* bounded between (−3, 0). The maximum isometric force was determined using a strength coefficient with a range of 0.5 to 2.5. The optimization process focused on minimizing the error between predicted and actual joint torques (computed via inverse dynamics) during the calibration procedure. This optimization task was achieved by employing a simulated annealing algorithm, which iteratives to refine the parameter values. The algorithm was executed until the average change in the objective function's value reached a tolerance level of 10^−5^.

### 2.3. NMS-BNN model

**Figure 4:**
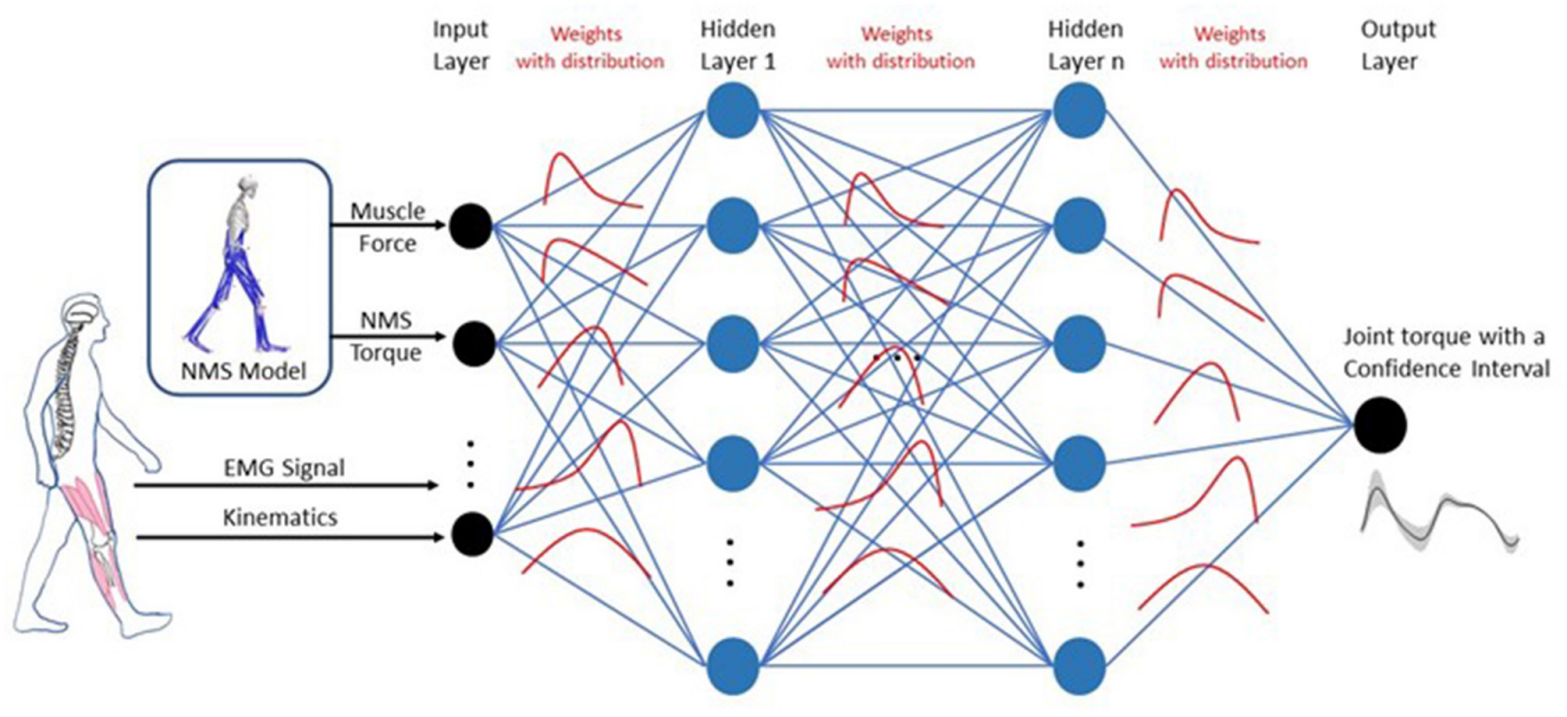
Architecture for the NMS-BNN model. The NMS-BNN models consist of an input neural layer, 3 hidden layers, and an output neural layer. The inputs, $\text{x}={\left[x_{1},x_{2},\ldots ,x_{m}\right]}^{T}$ where *m* = 21, were augmented with two types of features: (1) Muscle EMG signals and joint kinematics from a 3D motion capture system, and (2) Physical features such as muscle forces and NMS torque from an underlying NMS solver, to increase the model's accuracy by providing more information about the system being modeled. Each hidden layer has 40 neurons. The estimated knee torque with uncertainties bound was determined in the output layer. Weights are treated as probability distributions rather than as single-point estimates as in standard neural networks. These distributions are used to reflect the uncertainty in weights and predictions.

The NMS-BNN models consist of an input neural layer, 3 hidden layers, and an output neural layer. The inputs, $\text{x}={\left[x_{1},x_{2},\ldots ,x_{m}\right]}^{T}$ where *m* = 21, were augmented with two types of features (Figure 4): (1) Muscle EMG signals and joint kinematics (knee F/E angle) from a 3D motion capture system, and (2) physical features such as muscle forces and NMS torque from an underlying NMS solver, to increase the model's accuracy by providing more information about the system being modeled. Each hidden layer has 40 neurons. The estimated knee torque with uncertainties bound was determined in the output layer.

In BNNs, weights are treated as probability distributions rather than as single point estimates, as in standard neural networks (Figure 4). These distributions are used to reflect the uncertainty in weights and predictions. The posterior probability of weights, *P*(*W*|*X*), is computed using Bayes theorem as follows (Equation 6):


(6) $$\begin{array}{l} P\left(W|X\right)=\frac{P\left(X|W\right)P\left(W\right)}{P\left(X\right)} \end{array}$$

where *X* is the data, *P*(*X*|*W*) represents the likelihood of the data given weights *W*, and *P*(*W*) is the prior probability of the weights. The denominator, *P*(*X*), represents the probability of the data, which is obtained by integrating the likelihood of the data given weights and the prior probability of weights over all possible values of weights, represented by Equation (7):


(7) $$\begin{array}{l} P\left(X\right)=\int P\left(X|W\right)P\left(W\right)dW \end{array}$$

The BNN package TensorBNN, developed by Kronheim et al. (2022), was used in this study. The hyper-parameters of the BNN models were determined using a “coarse-to-fine” random search method (Bergstra and Bengio, 2012). During training, the mean square error was used as the loss function and a batch size of 32 was applied. Three hidden layers were included. Each hidden layer has 40 neurons with a tanh activation function. A Gaussian likelihood with a standard deviation of 0.1 was employed. Prior to sampling, the model was pre-trained using the AMSGrad optimizer with learning rates of 0.01, 0.001, and 0.0001, with a patience of 10. To obtain a point cloud of the posterior density of neural network parameters, Hamiltonian Monte Carlo (HMC) sampling was used to compute the likelihood function. HMC is a Markov chain Monte Carlo method that leverages a fictitious potential energy function derived from the posterior density of the neural network parameters. Numerical approximation was conducted using the leapfrog method, with the number of leapfrog steps and step size determining the distance traveled to the next proposed point. The number of steps for the HMC hyper-parameter sampler remained constant, while the step size was adapted using the Dual-Averaging algorithm based on the acceptance rate of the sample during 80% of the burn-in period. It is worth noting that selecting suitable values for the number of steps and step size can be challenging, and TensorBNN incorporates the parameter adapter algorithm to automatically optimize these parameters (Wang et al., 2013; Kronheim et al., 2022).

### 2.4. NMS-GP model

The NMS-GP model was developed using the same input data as the NMS-BNN model, which comprised experimentally obtained muscle signals and joint kinematics, as well as physical features such as muscle forces and NMS torque extracted from the underlying NMS solver. The NMS-GP model *f*(**x**) was specified by a mean function μ(**x**) and a covariance function *k*(**x**, **x**′), as expressed in equation (8) (Rasmussen, 2004).


(8) $$\begin{array}{l} f\left(\text{x}\right)\sim GP\left(\mu \left(\text{x}\right),k\left(\text{x},{\text{x}}^{{\;}^{\prime }}\right)\right) \end{array}$$

where the mean function μ(**x**) provides an estimate of the expected value of the model at a given input, while the covariance function, also referred to as the kernel function, quantifies the similarity between two inputs. The Gaussian process model offers various kernel functions to capture the underlying structure of data. Among them, the radial basis function kernel (RBF) is widely used due to its smoothness and infinite differentiability, as shown in Equation (9),


(9) $$\begin{array}{l} k\left(\text{x},{\text{x}}^{\prime }\right)=\exp\left(-\frac{|\text{x}-{\text{x}}^{\prime }{|}^{2}}{2l^{2}}\right) \end{array}$$

where *l* controls the length-scale of the kernel, and |**x**−**x**′| is the Euclidean distance between inputs **x** and **x**′.

Another popular kernel function is the Matern kernel, which is a generalization of the RBF kernel and is defined as Equation (10),


(10) $$\begin{array}{l} k\left(\text{x},{\text{x}}^{\prime }\right)=\frac{2^{1-\nu }}{\Gamma \left(\nu \right)}{\left(\frac{\sqrt{2\nu }}{l}|\text{x}-{\text{x}}^{\prime }|\right)}^{\nu }K_{\nu }\left(\frac{\sqrt{2\nu }}{l}|\text{x}-{\text{x}}^{\prime }|\right) \end{array}$$

where ν determines the smoothness of the kernel, *K*_ν_ is the modified Bessel function, Γ(ν) is the Gamma function, and *l* controls the scale of the kernel.

For modeling noise in data, the White Noise Kernel, as shown in Equation (11), is commonly used,


(11) $$\begin{array}{l} k\left(\text{x},{\text{x}}^{\prime }\right)=\sigma^{2}\delta \left(\text{x}-{\text{x}}^{\prime }\right) \end{array}$$

where σ^2^ is the noise variance parameter that determines the amplitude of the noise, and δ(**x**−**x**′) is the Dirac delta function. This function equals one when **x** = **x**′ and zero otherwise, ensuring that the kernel function is non-zero only at the diagonal of the input space.

The Linear kernel is another widely used kernel that models a linear relationship between the input and output variables. Specifically, it can be formulated as depicted in Equation (12):


(12) $$\begin{array}{l} k\left(\text{x},{\text{x}}^{\prime }\right)=\sigma^{2}{\text{x}}^{T}{\text{x}}^{\prime } \end{array}$$

where σ^2^ represents the variance parameter.

In Gaussian process modeling, the combination of different kernel functions can improve the performance of the model. In this regard, we selected a combination of Matern, White Noise, and Linear kernels after conducting extensive testing to obtain the most accurate predictions. The Matern kernel was used to capture non-linear patterns, while the White Noise kernel accounted for measurement errors and uncertainty, and the Linear kernel modeled linear relationships between the input and output variables. Hyperparameters such as length scale, signal variance, noise variance, and others were optimized during the training process to enhance the model's performance. In the Matern kernel, we set ν to 3/2, and the length scale was bounded between the range of (0.01, 200), with variance confined within the range of (10^−3^, 10^5^). Similarly, the White Noise kernel had a noise variance bounded between (0.03, 100), while the Linear kernel had a variance range of (10^−3^, 10^5^).

### 2.5. Knee exoskeleton

The knee exoskeleton hardware consists of a drive unit (Gen.1, Maxon, Switzerland), a 3D-printed thigh-shank frame, and thigh and shank straps (Orliman 94260, Spain). The drive unit features a brushless DC motor (EC90 flat), a MILE encoder with 4,096 counts per turn, a three-stage planetary gearbox with an 18-bit SSI absolute encoder, and an EPOS4 position controller. The drive unit is capable of providing a continuous torque of 54 Nm and a maximum torque of 120 Nm on a 20% duty cycle. The system can operate on a DC power supply ranging from 10 to 50vV and its actuation speed can reach up to 22 rpm.

### 2.6. Evaluation protocol

#### 2.6.1. Joint torque prediction

The prediction accuracy of the knee joint torque for NMS, NMS-GP, and NMS-BNN models was investigated. The uncertainty of the predicted torque by NMS-GP and NMS-BNN models was also analyzed. The prediction accuracy and uncertainty quantification was compared in five cases: *Gait*_*slow*_, *Gait*_*self*_, *Gait*_*fast*_, *SitToStand*, and *StandToSit*, which were trained using data from each movement separately. NMS-GP and NMS-BNN models were trained using 80% of the data and evaluated on the remaining data, while NMS models were calibrated using three trials of each movement and tested on the same data as NMS-GP and NMS-BNN models. The input data from each trial consisted of approximately 100 time-series data points and 21 dimensions.

Two prediction error metrics were evaluated: the Normalized Root Mean Square Error (NRMSE, *E*_*NRMS*_) and the Root Mean Square Error (RMSE, *E*_*RMS*_). A low prediction error indicated a high prediction accuracy. NRMSE was calculated by dividing the RMSE (between the predicted and actual torque) by the range of joint torque observed during the corresponding motion:


(13) $$\begin{array}{lll} E_{RMS} & = & \sqrt{\frac{1}{N}\overset{N}{\underset{n=1}{\sum }}{\left(y_{p,n}-y_{n}\right)}^{2}} \end{array}$$

(14) $$\begin{array}{lll} E_{NRMS} & = & \frac{E_{RMS}}{\left(y_{max}-y_{min}\right)}\times 100\% \end{array}$$

where *y*_*n*_ and *y*_*p, n*_ are the measured/actual and predicted torque respectively; and *y*_*min*_ and *y*_*max*_ are the minimum and maximum measured torque in corresponding movements. The RMSE and NRMSE were calculated for each subject and the average was obtained across eight subjects. The results section presents the average values of RMSE and NRMSE.

The normality of the data distribution was evaluated using Shapiro-Wilk tests (*p* < 0.05 significance level). To determine the differences among the NRMSEs and RMSEs estimated by the three approaches, pairwise comparisons were performed using either paired *t*-tests for normally distributed data or Wilcoxon signed-rank tests for non-normally distributed data, both with Bonferroni correction applied and significance level of *p* < 0.05.

The uncertainty of the predicted torque by NMS-GP and NMS-BNN models was quantified by using a 95% confidence level (CL), which means that there is a 95% probability that the true value of the function being modeled falls within the predicted interval. A high uncertainty value indicates low confidence in the predicted value.

#### 2.6.2. Exoskeleton assistive torque tracking performance

We also evaluate the tracking performance of the knee exoskeleton's assistive torque provided by the adaptive control framework during five daily activities by using the two metrics: NRMSE and RMSE (between desired and actual torque provided by the knee exoskeleton). The assistance level *A*_*L*_ of the knee torque provided by the adaptive control framework is adapted/determined by the uncertainties *U* quantified by the NMS-BNN model, as described by the following equation:


(15) $$A_{L}=\{\begin{array}{ll} 0.8 & \text{if }U<0.05\\ 0.6 & \text{if }0.05\leq U<0.1\\ 0.4 & \text{if }0.1\leq U<0.15\\ 0.3 & \text{if }0.15\leq U<0.2\\ 0.1 & \text{if }U\geq 0.2 \end{array}$$

## 3. Results

### 3.1. Joint torque prediction

**Figure 5:**
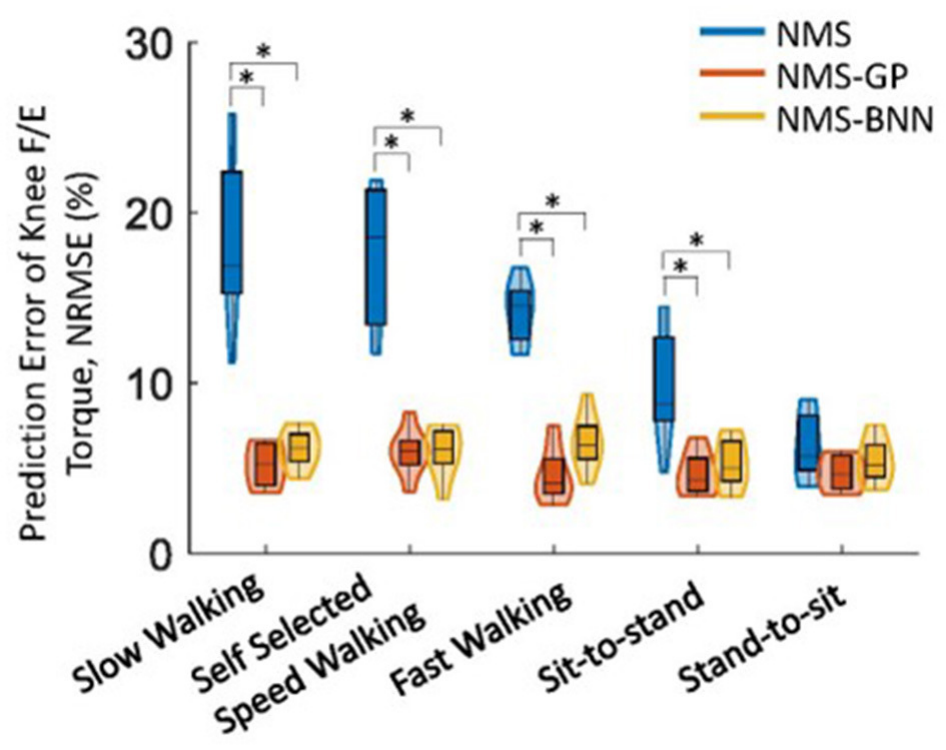
The distributions of NRMSE between estimated and measured/actual knee joint torques across subjects in NMS, NMS-GP, and NMS-BNN models during five daily activities. The violin plots depict the probability distributions of NRMSE using kernel density plots, and the box plots represent the minimum, lower quartile, median, upper quartile, and maximum values of NRMSE. A significant difference between any two models is indicated by an asterisk (*), based on paired *t*-test (for normally distributed data) or Wilcoxon signed-rank test (for non-normally distributed data) with Bonferroni correction.

**Figure 6:**
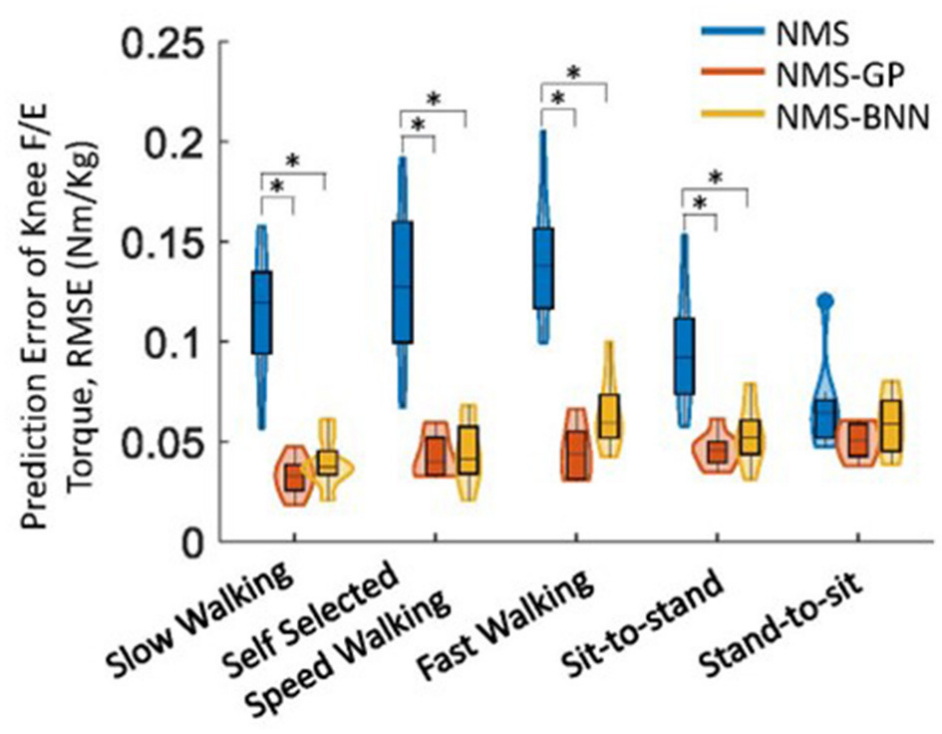
The distributions of RMSE between estimated and measured/actual knee joint torques across subjects in NMS, NMS-GP, and NMS-BNN models during five daily activities. The violin plots depict the probability distributions of NRMSE using kernel density plots, and the box plots represent the minimum, lower quartile, median, upper quartile, and maximum values of NRMSE. A significant difference between any two models is indicated by an asterisk (*), based on paired *t*-test (for normally distributed data) or Wilcoxon signed-rank test (for non-normally distributed data) with Bonferroni correction.

Overall, both NMS-BNN and NMS-GP models accurately predicted knee joint torque with relatively low error (RMSE: NMS-GP ≤ 0.05 Nm/kg, NMS-BNN ≤ 0.07 Nm/kg; NRMSE: NMS-GP ≤ 5.9%, NMS-BNN ≤ 6.8%). The errors were considerably lower than those of NMS models (RMSE: ≤ 0.14 Nm/kg, NRMSE: ≤ 18.3%, Figures 5, 6).

The NRMSE prediction error for the NMS-GP and NMS-BNN models was significantly lower than that of the NMS models in all cases, except the *StandToSit* case (*Gait*_*slow*_: *p* < 0.01 and *p* < 0.01; *Gait*_*self*_: *p* < 0.01 and *p* < 0.01; *Gait*_*fast*_: *p* < 0.01 and *p* < 0.01, *SitToStand*: *p* < 0.01 and *p* < 0.01, *StandToSit*: *p* = 0.08 and *p* = 1.45; Figure 5). Similar findings were also observed in the RMSE.

Among the NRMSE predicted by NMS models in five cases, the NRMSE in the *StandToSit* case was the lowest (≤ 7.2%). No significant differences were observed in the *StandToSit* case among NMS, NMS-GP, and NMS-BNN models (NMS: ≤ 7.2%, NMS-GP: ≤ 5.5%; NMS-BNN: ≤ 6.8%).

**Figure 7:**
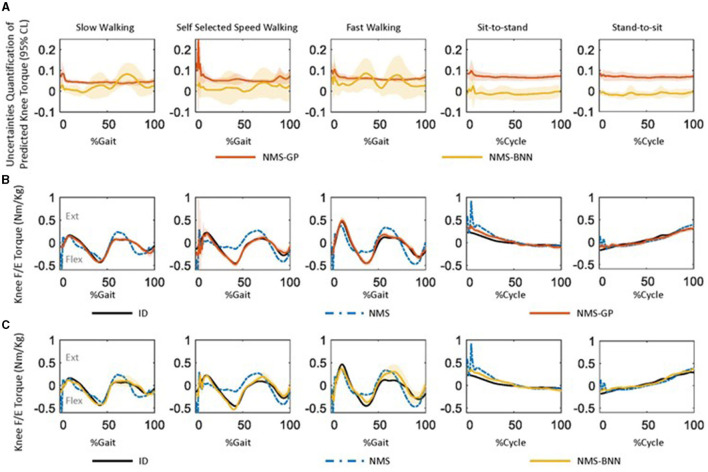
**(A)** The uncertainty quantification of predicted knee joint torque by the NMS-GP and NMS-BNN models across subjects during five daily activities, as the mean ± 1 standard deviation of all subjects. The uncertainty was quantified using a 95% confidence level, meaning that there is a 95% probability that the true value falls within the predicted interval. A high uncertainty value indicates low confidence in the prediction. **(B)** One example of measured knee flex/extension torques (Nm/kg) by inverse dynamics (ID) and predicted values by both NMS and NMS-GP models during five daily activities. The standard deviation in the NMS-GP models highlights the uncertainties from the expected mean value. **(C)** One example of predicted knee flex/extension torques by NMS-BNN models was presented and compared with the same example from ID and NMS models in **(B)**. The standard deviation in the NMS-BNN models also highlights the uncertainties from the expected mean value.

Both the NMS-GP and NMS-BNN models had relatively high uncertainties in the predicted knee torque at the beginning of each movement, particularly in the *Gait*_*self*_ case (Figure 7A). In the NMS-GP model, high uncertainties were observed during terminal stance and terminal swing in the *Gait*_*self*_ case. On the other hand, the NMS-BNN model had high uncertainties during terminal stance, initial swing, and terminal swing in all gait speeds.

The predicted torque by the NMS models had a poorer agreement with the measured torque compared to the NMS-GP and NMS-BNN models (Figures 7B, C). Relatively high offsets were observed at the beginning of each movement in NMS models.

### 3.2. Exoskeleton assistive torque tracking performance

**Figure 8:**
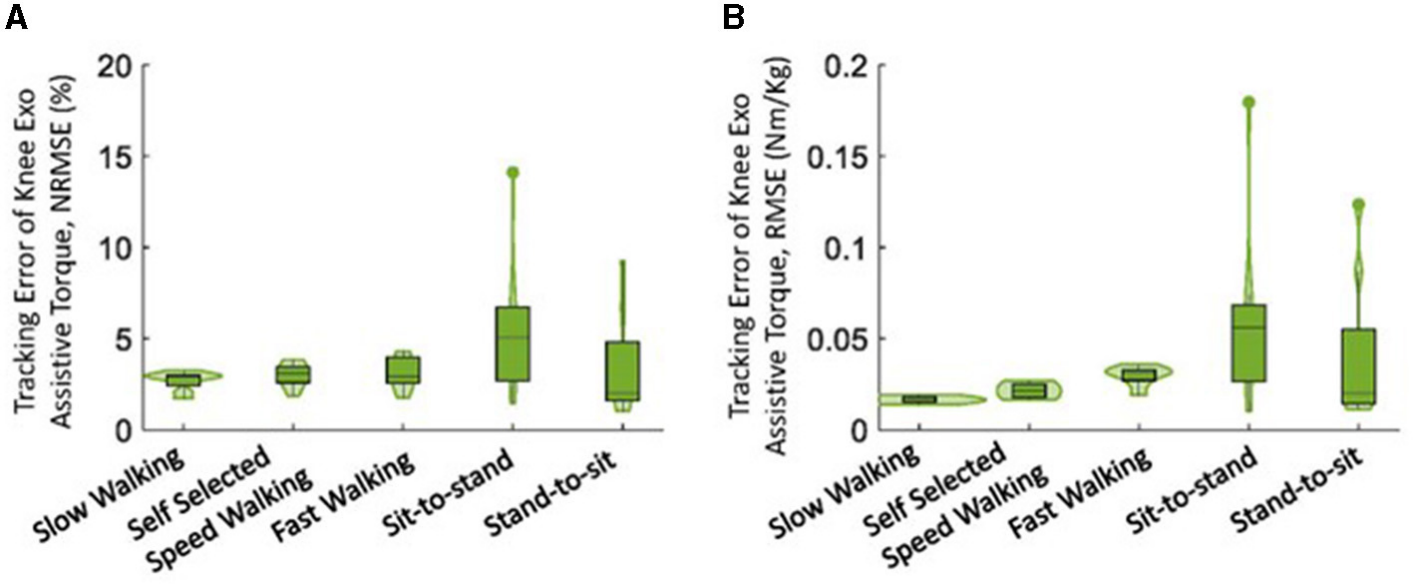
The tracking error of the knee exoskeleton's assistive torque provided by the adaptive control framework during five daily activities, presented as **(A)** NRMSE and **(B)** RMSE. The violin plots illustrate the probability distributions of prediction error through kernel density plots, and the box plots depict the minimum, first quartile, median, third quartile, and maximum values of the prediction error.

Overall, the knee exoskeleton accurately provided the required assistive torque with relatively low error (RMSE: ≤ 0.06 Nm/kg, NRMSE: ≤ 5.6%, Figure 8). Among the five movements, the NRMSE was evenly distributed among all subjects for walking movements, while one outlier was noted in both sit-to-stand and stand-to-sit movements. The sit-to-stand movement had the highest tracking error among the five movements.

**Figure 9:**
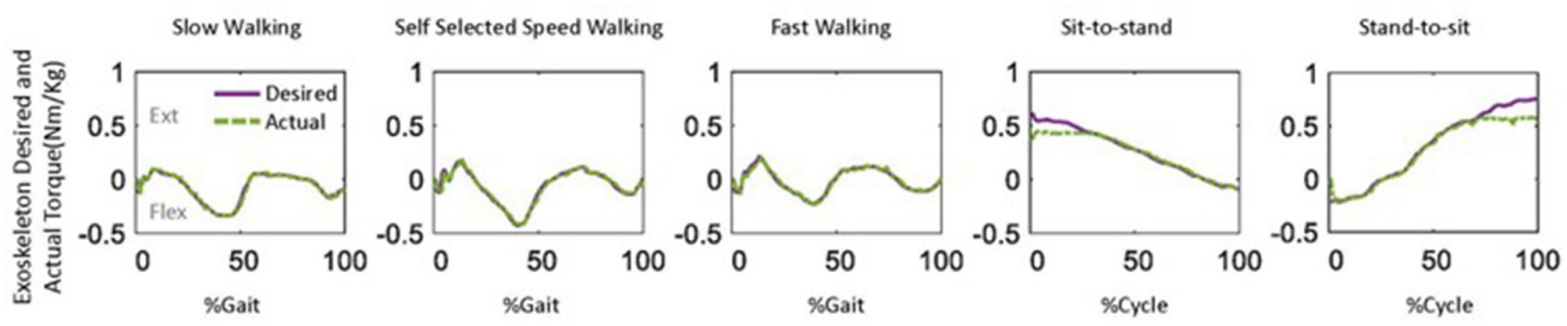
One example of the desired and actual assistive torque provided by the knee exoskeleton during five daily activities.

Generally, the actual assistive torque provided by the knee exoskeleton matched the desired torque well (Figure 9). However, it is important to note that limited torque was provided at the start of the sit-to-stand movement. Additionally, relatively low assistive torque was observed during the terminal stance of fast walking compared to self-selected speed walking.

## 4. Discussion

We developed an NMS-BNN-based adaptive control framework for a knee exoskeleton using muscle EMG signals and joint kinematics. We also compared the predictions with uncertainties from the NMS-BNN model with those from the NMS-GP model. We observed both NMS-BNN and NMS-GP models showed accurate predictions of knee joint torque with low error, outperforming traditional NMS models, indicating the benefits of incorporating NMS features into machine learning models. High uncertainties, however, were observed at the beginning of each movement and at terminal stance and terminal swing in the self-selected speed walking in both NMS-BNN and NMS-GP models. The knee exoskeleton provided the desired assistive torque accurately, with a relatively low error. Lower levels of torque were observed during terminal stance in fast walking compared to self-selected walking speed. The level of assistive torque was determined and adjusted based on the uncertainty in the NMS-BNN predictions, promoting the safety of the exoskeleton-user interface.

Incorporating the user's physiological joint torque into exoskeleton control strategies has in recent years become feasible, and has vast potential to improve task performance and rehabilitation outcomes. Among common techniques for predicting joint torque, EMG-driven NMS models require expertise and complex calibration, whereas machine learning models are more accessible but considered as black boxes (Hoang et al., 2018; Ezati et al., 2019; Soleimani and Nazerfard, 2021). To improve prediction accuracy, ANNs have been integrated into NMS models, allowing the advantages of both approaches to be leveraged. However, ensuring the safety and efficiency of exoskeleton control is also crucial, particularly when using predicted torque as inputs for the exoskeleton. To address this, this study integrated NMS with machine learning models with uncertainty quantification for joint torque prediction. As mentioned earlier, Both BNNs and GP models can provide predictions with associated uncertainties. While BNN models incorporate Bayesian methods to quantify the uncertainty in predictions, GP models are based on Gaussian processes and provide a probabilistic model for predictions with uncertainties. In the current study, we also compared the predictions with uncertainties between NMS-BNN and NMS-GP models. We found both NMS-BNN and NMS-GP models accurately predicted knee joint torque with relatively low error (RMSE: NMS-GP ≤ 0.05 Nm/kg, NMS-BNN ≤ 0.07 Nm/kg; NRMSE: NMS-GP ≤ 5.9%, NMS-BNN ≤ 6.8%), and were found to be superior to traditional NMS models (RMSE ≤ 0.14 Nm/kg, NRMSE ≤ 18.3%). The results are attributed to the addition of machine learning layers, which further train the model by minimizing the error between measured and predicted joint torque.

The quantification of uncertainty by either the NMS-BNN or NMS-GP models can supply the exoskeleton controller with valuable data for decision-making, which could enhance safety in the exoskeleton-user interaction. For instance, we observed high uncertainties at the beginning of each movement in both NMS-BNN and NMS-GP models (Figure 7). This is likely due to the physical characteristics adopted from NMS models, which show a noticeable offset at the start of each movement. In NMS models, two prior time steps of neural activation from each MTU are required to calculate muscle neural activation (Zhang et al., 2022). At the beginning of a cycle, these past two neural activation values are not yet obtainable and are approximated using EMG signals from two previous time steps, potentially leading to initial inaccuracies in predicted torque. Furthermore, high uncertainties were observed during the terminal stance and terminal swing in self-selected speed walking in both NMS-BNN and NMS-GP models. This may be attributed to the of transitions between the stance and swing phases of gait.

The knee exoskeleton provided the desired assistive torque accurately, with a relatively low error (RMSE: ≤ 0.06 Nm/kg, NRMSE: ≤ 5.6%, Figure 8). The assistive torque was achieved through current control in the motor, a widely used closed-loop control technique (Zhang et al., 2018; Azocar et al., 2020; Nuckols et al., 2021). The current control system aims to maintain a consistent current in the motor, even as its speed and load conditions vary. Precise control over the motor's torque production can be achieved through current control, though accuracy may be influenced by factors such as the quality of current sensing and the speed of the control loop's response. To estimate output torque, the control system uses the measured current as feedback, as the current is proportional to the torque produced by the motor (Azocar et al., 2020). This allows the control system to determine the amount of torque produced and adjust the exoskeleton accordingly. We observed lower levels of torque during the terminal stance of fast walking compared to self-selected walking speed (Figure 9). This discrepancy may be due to the increased uncertainties present during fast walking, which in turn led to a lower level of assistance torque being assigned according to our control strategy (Equation 15). It is worth noting that an outlier was observed in the sit-to-stand and stand-to-sit movements (Figure 8). This deviation may be attributed to the limited torque capacity of the exoskeleton at the beginning of the sit-to-stand movement and at the end of the stand-to-sit movement (Figure 9).

This study focused on evaluating the feasibility of the NMS-BNN framework by implementing a basic current control strategy. The objective was to assess the overall viability of the framework. However, future studies are necessary to investigate more advanced control techniques, such as impedance control. The current control strategy may result in less smooth assistive torque. Therefore, in future studies, we recommend incorporating an improved control strategy that takes into account both uncertainties and the closest points of predicted torque to enhance the smoothness and improve the user-exoskeleton interface. Furthermore, while our current study centers on the knee joint, it is important to note that the approach can be adapted and extended to other joints as well. Additionally, it is worth mentioning that the maximum torque that can be generated by the system is 54 Nm, which may also impact the smoothness of the assistive torque. Thus, this should be considered in future control strategies. It should be noted that this study did not involve testing the performance of the NMS-BNN-based adaptive framework on real users for practical applications. Further research is essential to address this issue and ascertain the practicality of the framework.

## 5. Conclusion

In this study, we proposed an NMS-BNN-based adaptive control framework for a knee exoskeleton that uses muscle EMG signals and joint kinematics. The NMS-BNN model combines a traditional NMS model with modern machine learning techniques and includes uncertainty quantification. The proposed framework also measures uncertainty in predictions and incorporates it into the control design to ensure safety of the exoskeleton-user interface. We also compared the performance of the NMS-BNN model to an NMS-GP model, which also predicts uncertainties. Detailed information relating to how to combine traditional models with machine learning models with uncertainties can provide useful guidance for designing exoskeleton control strategies.

## Data availability statement

The raw data supporting the conclusions of this article will be made available by the authors, without undue reservation.

## Ethics statement

The studies involving humans were approved by Swedish Ethical Review Authority. The studies were conducted in accordance with the local legislation and institutional requirements. The participants provided their written informed consent to participate in this study. Written informed consent was obtained from the individual(s) for the publication of any potentially identifiable images or data included in this article.

## Author contributions

LZ: Conceptualization, Data curation, Formal analysis, Investigation, Methodology, Software, Validation, Visualization, Writing—original draft, Writing—review and editing. XZha: Writing—review and editing, Formal analysis, Methodology, Investigation. XZhu: Methodology, Supervision, Writing—review and editing. RW: Methodology, Supervision, Writing—review and editing. EG-F: Conceptualization, Data curation, Funding acquisition, Methodology, Project administration, Resources, Supervision, Writing—review and editing.
